# Special Issue “Plant-Sourced Functional and Bioactive Compounds—Nature’s Blueprint for Improving Human Well-Being”

**DOI:** 10.3390/ijms27209142

**Published:** 2026-10-15

**Authors:** Dasha Mihaylova, Aneta Popova

**Affiliations:** 1 Department of Microbiology and Biotechnology, University of Food Technologies, 4000 Plovdiv, Bulgaria; 2 Department of Biochemistry and Nutrition, University of Food Technologies, 4000 Plovdiv, Bulgaria

Plants remain an enduring, irreplaceable focus of global scientific inquiry. They serve as an exceptionally rich reservoir of evolutionarily optimized bioactive substances with the potential to fundamentally improve human health, mitigate the onset of chronic metabolic and degenerative disorders, and enhance holistic well-being [1]. Their immense and intricate chemical diversity—comprising complex secondary metabolites such as structurally diverse polyphenols, multi-ringed flavonoids, bioactive alkaloids, and volatile terpenoids—continues to offer unprecedented, virtually inexhaustible opportunities for the discovery of next-generation functional food ingredients, targeted therapeutic agents, and innovative biomedical nanoplatforms [2]. Realizing this full translational potential from bench to bedside, however, requires rigorous, high-throughput molecular investigation that moves far beyond historical empirical observation. Contemporary molecular science demands not only a comprehensive, mechanistic elucidation of cellular efficacy and multi-target pharmacodynamics via network pharmacology and molecular docking [3], but also a meticulous quantification of intrinsic cyto- and genotoxicity, dose-dependent pharmacokinetic behaviors, and systemic regulatory safety gaps, thereby ensuring that nature-derived chemical entities can be integrated into modern clinical medicine safely, predictably, and with verified scientific confidence [4].

Since antiquity, the plant kingdom has served as the foundational cornerstone supporting human nutrition, protective clothing, and traditional healthcare frameworks across diverse global civilizations. In the contemporary era, this longstanding ethnobotanical and ethnopharmacological legacy has catalyzed intense multi-disciplinary interest toward a broad, validated spectrum of biological profiles, including potent free-radical-scavenging antioxidant capacities, precise anti-inflammatory cascades, targeted antitumor activities, and robust antimicrobial strategies spanning antibacterial, antiviral, antifungal, and antiparasitic mechanisms, alongside documented analgesic, antidiabetic, anti-atherogenic, antiproliferative, cardioprotective, and neuroprotective effects. At the molecular level, recent research has demonstrated that these diverse phytochemical ligands orchestrate their therapeutic effects by precisely targeting and modulating central cellular survival networks. Specifically, plant metabolites have been shown to directly intervene in the crosstalk between the Keap1-Nrf2/ARE cytoprotective pathway and the NF-κB pro-inflammatory axis, while concurrently regulating downstream kinase cascades including the mitogen-activated protein kinase (MAPK) and the PI3K/Akt/mTOR signaling networks [5,6].

At the same time, raw plant materials and unrefined botanical extracts inherently present complex biosecurity, pharmacological, and toxicological vulnerabilities; these include localized dermal reactions, acute systemic allergenicity, and hazardous contamination risks from heavy metals, synthetic pesticides, or pathogenic mycotoxins. The core mandate of modern phytochemistry and molecular biology is therefore to evaluate this accumulated traditional knowledge through a critical, highly evidence-based lens. By strategically leveraging advanced analytical platforms—such as high-resolution mass spectrometry, nuclear magnetic resonance, transcriptomics, and computational metabolomics—researchers can now decode precisely how specific phytochemical ligands modulate complex intracellular signaling networks and epigenetic markers, effectively confirming or refuting historical therapeutic claims [7]. Despite monumental technological leaps in green extraction methodologies (such as supercritical fluid and ultrasound-assisted extractions), coupled with artificial intelligence-driven drug discovery pipelines [8], an overwhelming majority of the global plant metabolome remains entirely unmapped and structurally uncharacterized, presenting both a formidable methodological challenge and a profound, high-impact opportunity for the international scientific community.

Mihaylova et al. [9] investigated the valorization potential of peach peels from eight Bulgarian peach varieties, characterizing their GC–MS profiles, free and bound phenolic compounds, and corresponding antioxidant, antimicrobial, and enzyme-inhibitory activities. Their findings highlight the potential of fruit-processing byproducts as sources of bioactive compounds and support their sustainable valorization in food and other functional applications.

The contributions to this Special Issue illustrate the breadth of contemporary phytochemical research. Lomiwes et al. [Contribution 1] investigated the relationship between berry consumption and cognition. Their findings indicate that berry phytochemicals may support spatial learning and memory and may help protect against age-related cognitive decline. The observed effects may involve antioxidant-mediated neuroprotective pathways and modulation of the hypothalamic–pituitary–adrenal axis.

Zanotti et al. [Contribution 2] examined plant-derived nanovesicles as promising alternatives to mammalian exosomes. These vesicles may offer reduced immunogenicity, improved bioavailability, and the added value of beneficial plant metabolites. Their translation into affordable therapeutic platforms nevertheless depends on the robust characterization of their molecular structure and cargo, as well as reliable methods for obtaining sufficiently large and homogeneous nanovesicle populations.

Martínez-Lobos et al. [Contribution 3] evaluated the abiotic stress experienced by *Baccharis macraei* Hook. & Arn. (Asteraceae) growing near an industrial complex. The study assessed the species’ physiological and metabolic status through its extracts and isolated compounds, together with photosynthetic capacity, metal content and production, antioxidant activity, and cytotoxicity against tumorigenic cell lines. This integrated approach demonstrates the value of connecting environmental exposure with phytochemical composition and biological response.

The practical potential of plant-derived products is further demonstrated by Pereira Filho et al. [Contribution 4]. Their team evaluated the acaricidal activity of the essential oil of *Piper aduncum* and its principal component, dillapiole, against larvae of *Amblyomma sculptum*, a tick species whose preferred hosts include equids and capybaras. By establishing lethal concentration values and examining effects on tick enzymes, the study provides insights into possible mechanisms of action and a basis for the future development of plant-derived acaricidal formulations.

*Melissa officinalis* (lemon balm), a source of flavonoids, phenolic acids, and essential oils, is considered to possess neuroprotective and antidepressant properties. Its possible benefits for sleep may be partly associated with rosmarinic acid and its modulation of γ-aminobutyric acid transaminase activity. Oliveira et al. [Contribution 5] reported that, in comparison with citalopram, lemon balm improved quality of life metrics, including sleep quality, and concluded that it may support cognition and sleep. They also emphasized that stronger evidence is required. Future research should therefore employ larger, adequately powered randomized clinical trials, rigorous randomization and blinding, careful control of concomitant medication, lifestyle factors, and comorbidities, and, where possible, objective measures or biomarkers rather than relying solely on subjective assessments.

Törős et al. [Contribution 6] critically reviewed plant-based nanomaterials, particularly those produced from agricultural byproducts such as mango seeds, tomato skins, and orange peels. In the face of the growing challenge of antibiotic resistance, these materials represent a promising antimicrobial strategy because they can act against a broad spectrum of pathogens through mechanisms that include reactive oxygen species generation and microbial membrane disruption, while presenting a reduced likelihood of promoting resistance. Their wider application in food systems and clinical practice will require standardized production methods and further investigations of dose-dependent effects across diverse populations.

Liu et al. [Contribution 7] consolidated recent evidence on plant-originating small molecules and their derivatives with anti-glioma activity. Although these compounds act through diverse mechanisms and are often considered less toxic than synthetic drugs, natural origin does not guarantee safety. Adverse effects may occur, particularly at high doses or after prolonged use. Thorough preclinical and clinical evaluations are therefore essential to establishing toxicity profiles and supporting safe clinical application. Further research should optimize bioavailability, potency, and selectivity while developing strategies to mitigate toxicity, with the long-term aim of delivering safe and effective nature-derived treatments that improve the prognosis and quality of life of patients with glioma.

Mashabela et al. [Contribution 8] provided an overarching account of the mechanistic basis of cannabidiol as a potential anticancer agent. The reviewed evidence indicates that phytocannabinoids can exert antiproliferative, anti-invasive, and proapoptotic effects across multiple cancer types. Cannabidiol appears to act through receptor-dependent and receptor-independent mechanisms, with pathway specificity that varies among cell lines and suggests cancer-specific modes of action. Despite this mechanistic diversity, the reported outcomes commonly converge on cell-cycle arrest, autophagy, cell death, or combinations of these processes. Its multiple targets also create opportunities for combination therapies in which co-administration with other medicines may produce an enhanced therapeutic response. Collectively, these findings support the continued investigation of cannabidiol as a potential anticancer agent and as a possible sustainable complement or alternative to existing treatments.

Taken together, the articles in this Special Issue demonstrate the remarkable range of plant-sourced functional and bioactive compounds and the diversity of approaches used to investigate them. From cognitive health, sleep, and cancer research to antimicrobial materials, environmental stress, nanovesicle technologies, and control of disease vectors, these studies show how phytochemical knowledge can contribute to pressing health and societal challenges. They also reinforce the importance of linking chemical characterization with mechanistic, toxicological, preclinical, and clinical evidence.

This collection further highlights the immense potential of unexplored biodiversity and confirms that the field remains far from exhausted. Progress will depend on interdisciplinary research, reproducible methods, standardized production and characterization, and careful assessment of efficacy and safety. As this Special Issue concludes, it provides both a record of current advances and a foundation for future inquiry. Continued work can help bridge traditional nature-based knowledge and modern medicinal science, translating the complexity of plant chemistry into responsible and evidence-based solutions for human well-being.
